# Appropriate interventions for the prevention and management of self-harm: a qualitative exploration of service-users' views

**DOI:** 10.1186/1471-2458-7-9

**Published:** 2007-01-19

**Authors:** Megan Hume, Stephen Platt

**Affiliations:** 1 Research Unit in Health, Behaviour and Change, School of Clinical Sciences & Community Health, University of Edinburgh, Teviot Place, Edinburgh, EH8 9AG, UK

## Abstract

**Background:**

The engagement of service-users in exploring appropriate interventions for self-harm has been relatively neglected in comparison with clinical studies focusing on the management and prevention of self-harm. The purpose of this study was to investigate perceptions of interventions for self-harm (formal and informal, prevention and treatment) among people who have first-hand experience as a result of their own behaviour.

**Methods:**

Semi-structured interviews were undertaken with 14 patients admitted to hospital following a repeat act of self-harm. Data analysis was undertaken thematically, drawing broadly on some of the principles and techniques of grounded theory

**Results:**

The patients were a heterogeneous group with respect to their personal characteristics and the nature of their self-harm. Thirteen of the 14 patient accounts could be assigned to one or more of three overlapping experiential themes: the experience of psychiatric illness, the experience of alcohol dependency, and the experience of traumatic life events and chronic life problems. These themes were related to the nature of patients' self-harm and their experiences of, and attitudes towards, interventions for self-harm and their attitudes towards these. There was a clear preference for specialist community-based interventions, which focus on the provision of immediate aftercare and acknowledge that the management of self-harm may not necessarily involve its prevention. The findings generate the preliminary hypothesis that personal circumstances and life history are major influences on the choice of interventions for self-harm.

**Conclusion:**

This study attests to the importance of recognising differences within the self-harming population, and acknowledging patients' personal circumstances and life history. These may provide clues to the antecedents of their self-harm, and lead to more acceptable and appropriate treatments.

## Background

Clinical guidelines for the management of self-harm [1] highlight the need for primary and secondary care services to provide a thorough assessment of mental health and social needs, precipitating factors and the risk of further self-harm or suicide among self-harming patients with whom they come into contact. By implication, appropriate treatment responses will be sensitive to differences *between *self-harm patients: interventions should acknowledge "diverse populations and diverse service needs" [2]. With user-led evidence increasingly recognised in research and policy-making [3], it is to be expected that service-user perspectives of the treatments received following self-harm would have been thoroughly investigated. However, the current evidence base is heavily reliant on studies that have been carried out 'on' self-harm patients, with a focus on managing and preventing self-harm from a medical perspective. There is an extensive body of literature based on service-user testimonies, particularly available on the internet [4]. Although providing a valuable insight into users' experiences, these testimonies are not part of the formal evidence base. The NICE guidelines [1] recommended that " [a] study using an appropriate and rigorously applied qualitative methodology should be undertaken to explore user experiences of services."

This pilot study aims to make a modest contribution towards fulfilling this recommendation through an investigation of interventions for self-harm (formal and informal, prevention and treatment), as perceived by people who have first-hand experience as a result of their own behaviour. In view of the relative neglect of research focus on repetitive self-harm [5], we recruited patients who had harmed themselves at least once previously. Repetitive self-harm places a heavy burden on health and social services and society as a whole. Up to half of hospital admissions following self-harm are repeat episodes, and a history of repetitive self-harm is a key risk factor for suicide [6]. A single previous episode of self-harm is associated with high suicidal intent in a subsequent episode [7].

## Methods

### Recruitment and sampling

Patients were recruited following admission to the Edinburgh Royal Infirmary (ERI) after a repeat act of self-harm during June and July 2005. Every attempt was made to generate a sample which included the full range of hospital-treated self-harm experiences in this population. Males and females, aged 16–50 years, with a history of self-harm were sampled. Children under the age of 16 were excluded for ethical reasons, and adults over 50 years because self-harm is rare in this age group [8]. Inclusion in the study required at least one previous act of self-harm within the last three years, with or without hospital admission(s). The ward staff assessed the psychological stability and medical fitness of potential participants who met the above criteria. Patients with learning difficulties, cognitive impairment or who were medically unfit were excluded, in addition to habitual drug users following an overdose, due to difficulties in establishing self-harm intent. Patients were selected using quota sampling, with four 'quotas' (based on two age groups [16–29 years, 30–50 years] and sex). Seventeen patients were approached, of whom 14 participated in an interview.

### Data collection

Patients' accounts were elicited through face-to-face qualitative interviews, using a semi-structured interview guide. Questions were intended to stimulate descriptions of experiences, attitudes to and feelings about treatments and interventions, as well as aspects of patients' life circumstances which were related to their self-harm. We drew on the notion of 'seed categories'[9] in order to inform the topic guide on which the interview was based. This illustrates a non-rigid adherence to grounded theory, as knowledge of the literature is not recommended by many of the traditional grounded theorists, but was considered constructive in this instance. Informed by the literature review, these 'seed categories' included the antecedents of, and influences on self-harm, such as the nature of social support, stressful life circumstances and psychiatric illness. The literature review suggested that the interview should explore experiences and perceptions in connection with a wide range of informal and formal interventions. Questioning was flexible, to allow patients to raise issues which were important and relevant to them, and permitted the researcher to develop questions and themes not anticipated at the outset. The initial questions on personal details were easily answered, and encouraged the development of a rapport before proceeding to the more sensitive questions. The final question was always a form of "*is there anything else that you feel I should have asked, or you would like to add?*" This proved to be very valuable and revealed a number of important new lines of enquiry.

All patients had been admitted to the ERI following a repeat act of self-harm within the last 48 hours. Interviews were carried out in a private room near the Combined Assessment Unit, where the patients were located. The ERI is the only service provider in Edinburgh City for patients admitted to hospital following self-harm. The interviewer introduced herself as a student, emphasising that the interview was not a part of, or connected to, the patient's treatment. Interviews lasted no more than one hour, with an average length of about 40 minutes. Ethical approval was sought and obtained from the Lothian Research Ethics Committee.

### Data analysis

The audio-taped interviews were transcribed verbatim. Data analysis was undertaken thematically, drawing broadly on some of the techniques of grounded theory, such as 'open' and 'axial coding'. [10]. Data collection and analysis occurred concurrently, with repeated comparison of emerging ideas within the expanding dataset. This iterative relationship between data collection and analysis guided questions in subsequent interviews, and decisions about what to explore in subsequent analysis. Initial concepts and relationships of interest were illuminated by searching for the salient patterns and shared themes between and among the cases. This was followed by a more rigid organisation of concepts into more abstract categories or themes. For example, about mid-way through the analysis, emerging concepts tended to be grouped around contextual aspects of the patients' lives. This was the foundation for the subsequent themes based on patient characteristics. Deviant case analysis and subsequent interviews and analyses led to revision and refinement of these categories. The use of diagrams, as recommended by Lofland and Lofland [11], proved fruitful in making connections within the data, identifying links between categories and deviant cases. For each interview a 'mind-map' was produced to display and draw attention to preliminary categories, and to help identify conceptual linkages between categories. Although the 'seed categories' were important in the early stages to guide interviews and analysis, their influence waned as the data expanded and the analytic approach became more inductive.

## Results

### Patient characteristics

Six females and eight males, aged between 20 and 49 years, were interviewed. All patients had harmed themselves at least twice previously, many on several occasions. They reported engaging in a variety of self-harming behaviours over the past three years (table 1). Over half the patients engaged in more than one form of self-harm. Self-poisoning was the most frequent reason for their current admission, which was to be expected in a hospital-based sample. Self-injury was more frequently reported overall, but was less likely to result in admission, consistent with findings from previous research [12]. High levels of suicidal intent were rarely found: only five patients reported a desire to end their life in connection with their most recent self-harm or a prior act. All 14 patients described a disrupted family life (e.g. death, divorce, separation), four reported experiencing abuse (sexual or physical) in childhood, and six lived in temporary accommodation. All but one patient was unemployed (seven had been out of work for several years), and 10 had received no education after the age of 17 years. Twelve patients described a history of alcoholism and/or depression and/or drug abuse. Three reported that they were 'depressed', three that they had borderline personality disorder and one, bi-polar disorder, although two had not received a formal diagnosis. The characteristics of the sample as a whole did not differ significantly from those of repetitive self-harmers reported in the literature [5]. However, it was not the case that patients conformed to a single profile: heterogeneity within the sample was particularly notable.

**Table 1:** Nature of self-harm in past three years (as reported by patients).

***Nature of self-harm***	***N engaging in behaviour at least once***
*Self-poisoning*	
Paracetamol	3
Antabuse	2
Ibuprofen	1
Anti-depressants	2
Battery	1
Unknown	1
*Self-injury*	
Cutting	7
Burning	1
Biting	1
Jumping	2
Other	2

N> 14 as multiple acts are included for each patient.

### Accounts of personal circumstances: three themes

Throughout the interview certain questions were intended to develop a picture of the patients' personal circumstances, and how these were perceived to be related to their self-harm. Thirteen of the 14 patient accounts could be assigned to one or more of three overlapping experiential themes: the experience of psychiatric illness; the experience of alcohol dependency; and the experience of traumatic life events and chronic life problems. The remaining patient was difficult to classify. Although he reported experiences which reflected aspects of the above themes, the interview was short and strained, and it proved difficult to probe into many of his responses.

#### Psychiatric illness

The experience of psychiatric illness emerged as a recurring theme, at least in part, in the accounts of seven patients. This was not based on any independent or third-party diagnosis of illness, but patients' own reports of depression, borderline personality disorder and bi-polar disorder, as well as anxiety and agoraphobia. Consistent with the well-documented relationship between psychiatric illness and repetitive self-harm [13], it was not surprising that, for all seven, the experience of psychiatric illness was interwoven with their accounts of self-harm, and that their self-harm was seen as inextricably part, or symptomatic, of their illness. As in the study by Sinclair and Green [2], they viewed their self-harm as a consequence of illness.

"*When they told me I had depression, I could think, that's why I do it (self-harm). It sounds stupid, but that made me feel better*" [female [F], aged 35 years].

"*I've got this borderline personality disorder, and that's who I am, you know, it's my personality, so that's why it [self-harm] will never stop. What do they want me to do? Change my personality?*" [F, 20].

In the present study, six of the seven patients described their self-harm as a means to get support and attention, because of frustration about not receiving support for their illness, with self-harm a "*sure thing*" [F, 35] for being admitted to hospital. They also reported sometimes feeling a strong desire to be admitted, to escape the overwhelming and often uncontrollable emotions leading to self-harm.

#### Alcohol dependency

Five patient accounts highlighted the significance of alcohol in their history of self-harm. One patient was presently abstinent, while alcohol had been involved in the most recent self-harm of the other four. For three patients self-harm was frequently the culmination of a binge drinking session which could last several days. Their drinking habit, which was often traced back to adolescence, served as an outlet for escaping problems and painful emotions. Feelings of hopelessness and low self-esteem associated with alcohol dependency were common among these patients. Their chaotic lifestyles, as evidenced by difficulties in securing and keeping jobs and living in temporary accommodation, also contributed significantly to their self-harm. Four patients described losing contact with, or the support of, friends and family through their alcoholism. Relationships with their 'drinking buddies' were superficial and not mutually supportive. All five patients described the pressures of overcoming an alcohol addiction as a factor contributing to their self-harm, yet viewed abstinence as the route to managing or prevailing over the behaviour. In discussing the role of alcoholism on self-harm, two patients considered their excessive drinking as self-harming in itself:

"*If anything, it's the booze that's gonna kill me*" [Male [M], 37].

The use of alcohol was not exclusive to the patients with alcohol dependency. In fact, only four patients in the whole sample claimed they had not consumed any alcohol prior to their self-harm. Some explained that alcohol served as a means of "*Dutch courage*" [F, 20], to numb the pain, or claimed that their self-harm was a result of "being drunk".

#### Traumatic life events and chronic life problems

Five patients' accounts were strongly characterised by traumatic life events or chronic life problems, including physical and sexual abuse in childhood, the death of a parent or sibling in infancy, illness in the family and the experience of HIV. All explicitly linked their self-harm in some way to such experiences. Typically these narratives were characterised by hopelessness and a pessimistic future outlook, particularly with respect to their self-harm. Four of these patients had harmed themselves for many years, with their self-harm becoming progressively worse over time, and described their behaviour as a long-term coping mechanism. These patients frequently expressed feelings of shame and guilt about receiving support for their self-harm. Such feelings were particularly intense amongst women and those patients who had a difficult family life, and were reluctant to place an extra burden on the family. One patient (F, 21) described how her parents had "*enough to deal with*" over her mother's illness and another described how her children took priority:

"*I want them [sons] to see me as a normal person*" [F, 40].

### Experiences of interventions for self-harm

Patients' experiences of interventions for self-harm were strikingly diverse. Only one patient reported an absence of contact with any services or support (excluding the current admission), while the rest described numerous interventions in connection with their self-harm (table 2).

**Table 2:** Nature of interventions for self-harm. Number of patient reporting experience of an intervention, on at least one occasion in their lifetime.

***Intervention (formal/informal)***	***N reporting experience of intervention***
General hospital admission	14
Accident and Emergency	14
G P	13
Family member	9
Friend	8
Specialist psychiatric admission	5
Counsellor	5
Social/Support worker	5
Community psychiatric nurse (CPN)	4
Alcohol Counsellor	4
Samaritans	3
Support group	2
Sheltered housing warden	2
Dialectical behaviour therapy	2
Cognitive behaviour therapy	1
Psychodynamic therapy	1

N>14 as most patients had experienced multiple interventions

The desire or willingness to engage with a service or source of support for self-harm was not uniform. Two patients specifically expressed a reluctance to make contact with the services offered to them.

"*It's pointless, there's nothing they can do, you can't stop a self-harmer*" [F, 21].

"*Everything I've ever been given is useless, the whole thing's buggered up*" [M, 37].

Those who were unwilling to engage with services were more likely to have been harming themselves over a long period. In line with this, patients spoke of feeling they were "*beyond help*" [F, 21] or "*defeated*" [M, 34]. The unwillingness to seek support for self-harm was most strongly expressed by patients whose accounts were characterised by traumatic life events (especially in childhood) or chronic life problems (including coping with the consequences of childhood trauma), although not exclusively.

Ten patients voiced a greater degree of willingness to engage with a variety of services, sharing an aspiration to minimise their self-harming behaviour, and were more likely to remain in long-term contact with services.

"*I badly want to ... stop ... I've been asking for help, I'm willing to try anything*" [F, 26].

Those patients who reported a longer commitment to a particular intervention tended to recount feeling satisfied with this service. In contrast, experience of a large number of different interventions was associated with less commitment to, or perseverance with, any particular intervention. This was particularly true of those individuals with a history of alcohol dependency. Patients who expressed negative feelings about their experiences of interventions for self-harm frequently declared their awareness of their rights as a patient, as illustrated by comments such as:

"*I've told them ... I want a complaint form and I want to get *[hospital staff] *sacked*" [M, 37].

However, in contrast, four patients (all female) felt that they were not in a position to feel or demonstrate any dissatisfaction, and dwelled on feelings of guilt, linked to the self-inflicted nature of their injuries:

"*You feel like a fraud ... [there are] wards full of people who are not well, and you want to punish yourself even more because ... there is other people who need the space more than you*" [F,40]

### Ideal interventions

Patients were encouraged to imagine 'ideal' interventions for their self-harm. They were given written descriptions of several interventions – problem solving therapy, cognitive behaviour therapy, drug therapy (medicines), support groups and the use of an emergency card (for 24 hour professional support) – in order to stimulate opinions.

#### Immediate aftercare

The key intervention identified by patients was immediate after-care (i.e. following discharge from hospital). Seven patients reported their dissatisfaction at having to wait long periods of time for an appointment following a previous act of self-harm, with a counsellor, for example:

"*I had to wait 12 weeks. A lot can happen in 12 weeks. When the appointment came I was, like, I didn't really see the point*" [F, 20].

For some, particularly those without significant support from family and friends, this delay gave rise to a fear that they would repeat the self-harm while waiting for an appointment.

"*What I'm thinking is I'll be discharged, and I'll have to go back to this empty flat. Nothing has really changed for me, and I know I'll have to wait, you know, 'til it comes [appointment card]*" [F, 25].

Eight patients were supportive of the emergency card [14]. The idea of being able to contact a professional "*twenty-four seven*" [F, 26] was reassuring, because

"*Sometimes it's hard to find someone to talk to*" [F, 20].

However, four patients with experience of the emergency card or a similar emergency contact, such as the psychiatric emergency team (PET), reported negative encounters or feeling uneasy.

"*I don't want to be a nuisance. People who self-harm don't want to bring attention to themselves, simple as that*" [M, 37].

#### Community versus hospital-based support

The preference for support for self-harm delivered by specialists outside the hospital setting has been highlighted in the service-user literature [15], and has been identified as a key approach in the management of self-harm [1]. The distinction between hospital and community-based services, and the preference for the latter, emerged in the majority of interviews. For some, this was related to feelings of guilt about being in the hospital among other (more 'deserving') patients, and concern about being judged by staff and other patients. Those with multiple episodes of self-harm described feeling uncomfortable about repeatedly attending the same hospital and encountering the same staff:

"*The more you come in, the more they (staff) lose their tether w'you*" [F, 49].

For other patients, admission to hospital, particularly to a psychiatric ward, was frightening, heightening already intense feelings of being out of control:

"*I speak positively about it now, but back at the time is was terrible. Locked wards, psychopaths, they used straightjackets and straps*" [M, 34].

However, despite this preference for community-based services, some patients occasionally expressed a desire to be admitted to hospital. This was mentioned by seven patients as a chance to

"*get away from it all*" [F, 26]

and to

"*just be cared for*" [M, 34].

The preference for community-based care was also evident in patients' preferences for contact with certain professionals (such as community psychiatric nurses and social workers) who have specialist knowledge and experience with people who self-harm, and have the potential to build up long-term relationships with their clients. One patient described his most supportive encounter, with a hospital chaplain, contrasting this with his attitude of the medical staff:

"*The chaplain ... praying and stuff like that ... they're not in it for the money if you know what I mean ... they're mair [more] committed, duty bound to help through their faith and stuff*" [M, 39].

It was most difficult to elicit ideas about ideal interventions for self-harm from patients whose life-circumstances were characterised by alcohol dependency. When articulated, their preferences tended to be centred on the alcohol services, particularly the need to overcome their alcoholism in order to access support for their self-harm. One patient [M, 37] described a good relationship with a prison alcohol counsellor, but was frustrated by the lack of help for his alcohol problems following his release, which he deemed contributory to his self-harm.

Almost all patients expressed a desire for mutual support and shared understanding from others who have harmed themselves, yet only two patients had actually attended a support group, and none of the others had been offered this service. Four patients, who had unfavourable attitudes to support groups, reported negative previous experiences of social support, and, in the case of two patients, a fear of strangers and long-standing agoraphobia. It is notable that the role of friends or family as a source of support was not always consistent with the desire for non-hospital based services. Five patients described a friend or family member as the single greatest source of support in connection with their self-harm, more important than any other source:

"*My wife ... she's a diamond, if it wasn't for her I don't know what I'd do*" [M, 41].

"*If it wasn't for her [friend] I wouldn't be here now*" [F, 26].

Yet other patients described feeling uneasy about being supported by friends and, particularly, family. This was explained by feeling a burden on family and friends, with a preference for support from a specialist "*whose job it is*" [F, 25].

Although the hospital environment was not the favoured setting for support for self-harm, the majority of patients described the hospital staff very positively, as sympathetic and understanding:

"*The ambulance driver ... he came back from another job and just popped his head round. It was really really really good, something I really appreciated. And the nurses... they were really nice to me, and gave me a lot of sympathy ... one of them I smelt she's been smoking, and I really needed a smoke, and she said I'll sort you out later. They were just really nice to me*" [M, 22].

Only five patients spoke of a strong dissatisfaction with hospital staff, especially those working in accident and emergency. These patients admitted being drunk on admission, which inevitably strained the situation, although there was evidence that some felt differently in their present sober state. Frequent attenders expressed similar dissatisfaction. One patient on the 'frequent attenders' list' complained that:

"* [This] means you come in and get treated generally, but you can't see a psychiatrist [...] they say you come in too often [...] the more harm you do, the less help you get*" [M, 34].

#### Management versus prevention: the need to self-harm

Only four patients spoke of terminating their self-harm as their main concern. Instead, they expressed a preference for interventions which enabled them to feel in control of the behaviour. The expression of a 'need' to self-harm is perhaps not unexpected given that this was a sample of repetitive self-harmers. The clear distinction between management and prevention of self-harm was particularly evident in the accounts characterised by the experience of traumatic life events, with self-harm seen as an important coping mechanism for dealing with distress:

"*I made a pact with myself; I was going to overdose on Thursday. That's my way of getting through the week, making it easier*" [M, 34].

"*Self-harming is the only thing that releases my pressure. It is a way of coping with the self-hate I have of myself*" [F, 40].

Several patients were anxious to impress on their friends, family and, in some cases, professionals the importance of managing self-harm (rather than its prevention):

"*I don't want to stop cutting myself. It's what I do. The sooner they understand you can't stop a self-harmer, the better*" [F, 21].

## Discussion

### Main findings

This sample of repetitive self-harming patients had diverse experiences and attitudes relating to interventions following self-harm. Despite this diversity, however, there was a shared preference for specialist community-based interventions which focus on providing immediate aftercare and acknowledge an approach to managing self-harm in addition, and often in preference, to its prevention. The preference for community-based care is not surprising in light of the existing service-user literature. The findings support the appropriateness of the move towards the provision of this type of integrated support for people who self-harm [16].

Early in the course of data analysis it was noted that patients' personal circumstances and life history appeared to be strongly related to the nature of their self-harm and their attitudes towards appropriate interventions. For example, although most patients tended to report favourably on their encounters with hospital staff, there was a trend amongst the patients with alcohol dependency to be more dissatisfied with these encounters. On further questioning it emerged that these patients were drunk on arrival at the hospital, and they admitted that this strained the situation. Similarly, whilst they shared an overall preference to manage their self-harm within a community setting, some patients whose personal circumstances were characterised by the experience of psychiatric illness expressed a desire, on occasion, to be admitted to hospital. These findings generate the preliminary hypothesis that personal circumstances and life history are major influences on the choice of interventions for self-harm which are perceived to be both effective and appropriate.

This study confirms the importance of recognising and acknowledging heterogeneity within the self-harming population. The development of typologies of suicidal behaviours and self-harm is not a new pursuit. Several studies in the 1970s attempted to create classifications of people who self-harm [17,18], but these efforts were not of great clinical value and were gradually abandoned. The research focus consequently shifted towards clinical interventions and assessment of their effectiveness, often focusing on the commonalities, rather than the variations, of self-harm. Despite heavy investment in this area, evidence of effective secondary interventions for repetitive self-harm remains remarkably scarce [19]. This leaves scope for studies which seek to draw on the experiences and perceptions of service users to contribute to the development of alternative interventions. This is an ethical approach to evidence-based medicine [3]; rather than assessing interventions based purely in terms of efficacy, it acknowledges the appropriateness and acceptability of interventions to patients.

### Study limitations and implications for clinical practice and further research

In view of the relatively small scale of this research, the provisional nature of the findings would benefit from (dis)confirmation in a larger-scale study. Alternative sources of patient recruitment beyond the hospital, such as via a support group in the community, and/or the involvement of relatives of self-harming patients or service providers, would serve to elicit potentially different accounts which would help to refine or refashion the hypothesis generated in the course of this study. Further interviews with a new sample drawn from the relevant population would provide opportunity to test the empirical generalisability of the conclusions.

A certain element of social distance between the researcher and patients was evident in some interviews, arising from gender, accent, ethnicity and, perhaps most significantly, the absence of shared experiences (especially relating to self-harm). The fact that the patients were interviewed soon after the self-harm episode could be viewed as an important strength, as the interviewer was able to take advantage of participants' heightened awareness of the causes and consequences of their behaviour. However, although the patients were assessed as fit for interview, their accounts may have been coloured by the lingering effects (psychological and toxicological) of the recent self-harm event. It is acknowledged that the patients' stories cannot necessarily be taken at face value. Their accounts of their experiences may reflect their emotional state and be coloured by their motivations for taking part, such as the need to rationalise or make sense of their own behaviour. The reference to "straightjackets and straps" (see above) is unlikely to be factually true but may nonetheless provide a reliable indication of the subjective emotional distress associated with the experience of hospitalisation.

Multiple interviews would have facilitated the development of rapport, which was restricted by the use of a single interview and the hospital setting, and provided a partial solution to the problem of accessing people's private accounts. Without any reasonable opportunity to develop interpersonal trust, patients' narratives may be characterised by a 'public' perspective [20]. In particular, further interviews would have been advantageous in developing an understanding of patients' reasons for engaging and not engaging in services. Given the constraints on time available for fieldwork, however, the use of multiple interviews was not possible.

An additional preference would be to hold the interviews in a more neutral setting, outside the hospital. This might help patients to think about their experiences more broadly: in this study they tended to focus on hospital-based experiences, often requiring considerable prompting to consider interventions in other settings.

## Conclusion

We suggest that there is further potential to translate the findings of the present study, and of similar research which acknowledges the heterogeneity of people who self-harm, into specific and appropriate interventions, tailored towards individual needs [5]. Appreciation of the individual's life circumstances may help to uncover the antecedents of their self-harm, and has important consequences for adopting an intervention which is effective, appropriate and based on the assessment of need. For instance, support for self-harm which occurs alongside alcohol dependency should be provided via, or in close connection with, the specialist alcohol services. Similarly, where preference is expressed for support services outside the hospital environment, self-harm should be managed within community based services, which can better support the user's preference for developing a relationship of trust with the professional. The role of community-based care in the management of self-harm is expanding, as evidenced by community psychiatric care, outreach teams, home treatment and crisis care such as the Samaritans. The findings of this study serve to confirm the potential value and acceptability of these community-based approaches to managing self harm.

## Competing interests

The author(s) declare that they have no competing interests.

## Authors' contributions

SP conceived the study, and both authors were responsible for its design and coordination. MH carried out the interviews and data analysis, and drafted the manuscript. Both authors read and approved the final manuscript.

## Pre-publication history

The pre-publication history for this paper can be accessed here:


